# Can CRISPR gene drive work in pest and beneficial haplodiploid species?

**DOI:** 10.1111/eva.13032

**Published:** 2020-06-19

**Authors:** Jun Li, Ofer Aidlin Harari, Anna‐Louise Doss, Linda L. Walling, Peter W. Atkinson, Shai Morin, Bruce E. Tabashnik

**Affiliations:** ^1^ Department of Statistics University of California Riverside CA USA; ^2^ Department of Entomology Hebrew University of Jerusalem Rehovot Israel; ^3^ Department of Entomology University of California Riverside CA USA; ^4^ Department of Botany and Plant Sciences University of California Riverside CA USA; ^5^ Department of Entomology University of Arizona Tucson AZ USA

**Keywords:** CRISPR/Cas9, gene drive, genetic engineering, haplodiploid, pests, pollinators, sex‐linked

## Abstract

Gene drives based on CRISPR/Cas9 have the potential to reduce the enormous harm inflicted by crop pests and insect vectors of human disease, as well as to bolster valued species. In contrast with extensive empirical and theoretical studies in diploid organisms, little is known about CRISPR gene drive in haplodiploids, despite their immense global impacts as pollinators, pests, natural enemies of pests, and invasive species in native habitats. Here, we analyze mathematical models demonstrating that, in principle, CRISPR homing gene drive can work in haplodiploids, as well as at sex‐linked loci in diploids. However, relative to diploids, conditions favoring the spread of alleles deleterious to haplodiploid pests by CRISPR gene drive are narrower, the spread is slower, and resistance to the drive evolves faster. By contrast, the spread of alleles that impose little fitness cost or boost fitness was not greatly hindered in haplodiploids relative to diploids. Therefore, altering traits to minimize damage caused by harmful haplodiploids, such as interfering with transmission of plant pathogens, may be more likely to succeed than control efforts based on introducing traits that reduce pest fitness. Enhancing fitness of beneficial haplodiploids with CRISPR gene drive is also promising.

## 1 INTRODUCTION

Gene drive mediated by the clustered regularly interspaced short palindromic repeats/CRISPR‐associated protein 9 (CRISPR/Cas9) system has recently emerged as one of the most promising technologies for reducing the harm caused by insect vectors of disease, crop pests, and invasive species, as well as for enhancing the fitness of valued species (Champer et al., 2018; Eckhoff, Wenger, Godfray, & Burt, 2017; Esvelt, Smidler, Catteruccia, & Church, 2014; Gantz et al., 2015; Godfray, North, & Burt, 2017; Grunwald et al., 2019; Kyrou et al., 2018; Rode, Estoup, Bourguet, Courtier‐Orgogozo, & Débarre, 2019; Scott et al., 2018). Cas9 is an endonuclease whose target site is determined by an independently expressed guide RNA (gRNA) via a 20‐nucleotide protospacer sequence (Noble, Olejarz, Esvelt, Church, & Nowak, 2017). Because Cas9 can target almost any sequence that is followed by a protospacer adjacent motif (PAM), RNA‐guided gene drive elements can be constructed by inserting a suitable sequence encoding Cas9 and one or more gRNAs (Hammond et al., 2016; Noble et al., 2017). In principle, individuals with the desired genotype can be engineered in the laboratory and released to spread this genotype in wild populations. Like some other gene drives, this approach works via segregation distortion, where heterozygotes transmit a desired allele at a frequency higher than the 50% expected under the Mendelian inheritance (Drury, Dapper, Siniard, Zentner, & Wade, 2017). In heterozygotes, CRISPR homing gene drives convert wild‐type alleles to driver alleles via cleavage and homology‐directed repair (Champer et al., 2018).

In parallel with empirical work, mathematical modeling and computer simulations have been essential for evaluating the feasibility, limitations, risks, and benefits associated with CRISPR gene drives (Champer et al., 2020; Deredec, Burt, & Godfray, 2008; Deredec, Godfray, & Burt, 2011; Eckhoff et al., 2017; Godfray et al., 2017; Hammond et al., 2016; Noble et al., 2017; Rode et al., 2019; Unckless, Clark, & Messer, 2017; Unckless, Messer, Connallon, & Clark, 2015). This theoretical work has identified evolution of resistance to the drive as a major limitation and greatly advanced understanding of the expected evolutionary trajectories of CRISPR gene drives in diploid species, particularly mosquito vectors of human disease. By contrast, little is known about the evolutionary dynamics of CRISPR gene drive in haplodiploid species, despite recognition of their considerable importance and the potential for using CRISPR gene drive to manage them (McLaughlin & Dearden, 2019; Rode et al., 2019; Scott et al., 2018).

In haplodiploids, unfertilized eggs yield haploid males and fertilized eggs yield diploid females. Haplodiploids include some of the world's most devastating pests of crops and forests, such as whiteflies in the *Bemisia tabaci* species complex, thrips (Thysanoptera), spider mites (Tetranychidae), and tree‐killing bark beetles (Scolytinae) (Biedermann et al., 2019; De Barro, Liu, Boykin, & Dinsdale, 2011; He, Guo, Reitz, Lei, & Wu, 2020; Macfadyen et al., 2018; Normark, 2003; Van Leeuwen, Tirry, Yamamoto, Nauen, & Dermauw, 2015). Also, more than 100,000 species of Hymenoptera are haplodiploid, including bees, ants, sawflies, and wasps that are critically important as pollinators, pests, natural enemies of pests, and invasive species in native habitats (McLaughlin & Dearden, 2019; Normark, 2003). Web of Science topic searches identified over 1,000 publications with the term “haplodiploid*” and 633 with “gene drive,” but none with both terms. Although the search with both terms missed a few papers that briefly mention gene drive for haplodiploids (McLaughlin & Dearden, 2019; Rode et al., 2019; Scott et al., 2018), the results reflect the limited attention this topic has received. Moreover, analysis of CRISPR gene drives in haplodiploids can elucidate the functionally equivalent gene drives at sex‐linked loci in diploids. In particular, drives affecting X‐linked loci are well known from experimental work with *Drosophila*, but modeling of their evolutionary trajectories has been limited (Champer et al., 2018).

**FIGURE 1:**
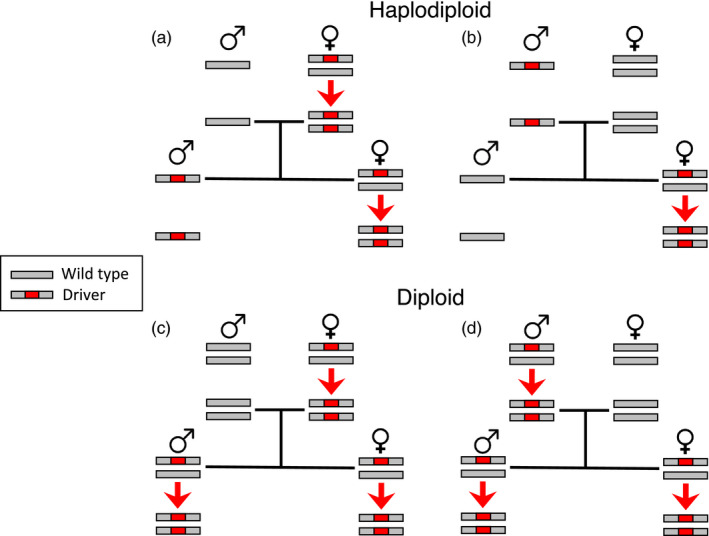
CRISPR‐mediated gene drive in haplodiploid and diploid species. Conversion from the wild‐type to driver allele (red arrow) occurs only in heterozygotes harboring one copy of each allele. In haplodiploids (a and b), conversion occurs only in females, which slows the spread of the driver allele relative to diploids (c and d), where conversion occurs in both sexes. In this example, the conversion rate is 100% (*c* = 1) and the driver allele has no fitness cost (*s = *0). In the parents (top row in each panel), the initial frequency of the driver allele (*q_0_*) is 0.5 for males and 0.25 for females for the haplodiploid (a and b pooled), and 0.25 for both sexes for the diploid (c and d pooled). The final driver allele frequency in the offspring after conversion (bottom row in each panel) is 0.5 for males and 1.0 for females for the haplodiploid (a and b pooled) versus 1.0 for both sexes for the diploid (c and d). The process illustrated for haplodiploids also applies to sex‐linked genes in diploids

The differences in inheritance between haplodiploids and diploids (and between sex‐linked and autosomal loci in diploids) are likely to affect outcomes of CRISPR gene drive in wild populations. First, conversion of wild‐type/driver allele heterozygotes to driver allele homozygotes can occur only in diploid females of haplodiploids versus both sexes in diploids (Figure 1). Second, selection acts directly on recessive alleles in haploid males, whereas such alleles are shielded from selection in heterozygous diploids (Crowder & Carrière, 2009). Thus, Rode et al. (2019) hypothesized that for CRISPR gene drive, deleterious alleles intended to suppress populations of harmful species would spread less readily in haplodiploids than diploids, but beneficial alleles in “rescue drives” intended to enhance or restore populations of valued species would spread more readily in haplodiploids than diploids. Here, we tested these hypotheses using population genetic models to compare the evolutionary dynamics of CRISPR gene drive in haplodiploids versus diploids. We discovered that in haplodiploids relative to diploids, as predicted, conditions favoring spread of deleterious alleles by CRISPR gene drive are more restricted, the rate of spread is slower, and resistance to the drive evolves more readily. Contrary to the prediction by Rode et al. (2019), beneficial alleles also spread slower in haplodiploids than diploids. However, the spread of alleles that cause little fitness cost or enhance fitness was not greatly hindered in haplodiploids relative to diploids.

## 2 METHODS

### 2.1 Modeling approach

We used deterministic, single‐locus models with discrete generations, a 1:1 sex ratio, and random mating to compare evolutionary dynamics between haplodiploids and diploids. CRISPR‐mediated conversion of wild‐type to driver alleles in heterozygotes occurred in the zygotes or in the adult gonads (germline). In both scenarios, the driver alleles generated from conversion in parents were inherited by offspring (Figure 1). Nine previous papers with evolutionary models of CRISPR gene drive in diploids examined conversion that occurred in zygotes (Drury et al., 2017; Unckless et al., 2015,2017), germline (Champer et al., 2020; Eckhoff et al., 2017; Hammond et al., 2016; Rode et al., 2019), or either (Deredec et al., 2008; Hammond et al., 2017).

### 2.2 Models without resistance to drive

In our basic models without resistance to the gene drive and conversion in zygotes, we used equation 8 of Deredec et al. (2008) for diploids and modified this equation to represent haplodiploids. We let *c* be the proportion of wild‐type alleles in wild‐type/driver allele heterozygotes converted to the driver allele, *s* the fitness cost of the driver allele, and *h* the dominance of the fitness cost of the driver allele in heterozygotes. For haplodiploids in generation *t*, we denote the frequencies of the wild‐type and driver alleles in males by *p_M,t_* and *q_M,t_* and their counterparts in females by *p_F,t_* and *q_F,t_*. In generation *t + 1*, these frequencies are as follows:

(1) $$p_{M,t+1}=\frac{p_{F,t}}{p_{F,t}+q_{F,t}\left(1-s\right)}\;\text{and}\;q_{M,t+1}=\frac{q_{F,t}\left(1-s\right)}{p_{F,t}+q_{F,t}\left(1-s\right)},$$

(2) $$p_{F,t+1}=\frac{p_{F,t}p_{M,t}+\frac{1}{2}\left(1-c\right)\left(1-hs\right)\left(p_{F,t}q_{M,t}+q_{F,t}p_{M,t}\right)}{w_{t}},$$

and

(3) $$q_{F,t+1}=\frac{\left(1-s\right)q_{F,t}q_{M,t}+\left\{c\left(1-s\right)+\frac{1}{2}\left(1-c\right)\left(1-hs\right)\right\}\left(p_{F,t}q_{M,t}+q_{F,t}p_{M,t}\right)}{w_{t}},$$

where

(4) $$\begin{array}{cc} w_{t} & =p_{F,t}p_{M,t}+\left\{c(1-s)+(1-c)(1-hs)\right\}\left(p_{F,t}q_{M,t}+p_{M,t}q_{F,t}\right)\\ & \;+\left(1-s\right)q_{F,t}q_{M,t}. \end{array}$$

In our basic models without resistance to the gene drive and conversion occurring in the adult gonads (germline), we used equation 1 of Deredec et al. (2008) for diploids and modified this equation to represent haplodiploids. In generation *t + 1*, the frequencies of the wild‐type and driver alleles in males and females are as follows:

(5) $$p_{M,t+1}=\frac{p_{F,t}}{p_{F,t}+q_{F,t}\left(1-s\right)}\;\text{and}\;q_{M,t+1}=\frac{q_{F,t}\left(1-s\right)}{p_{F,t}+q_{F,t}\left(1-s\right)},$$

(6) $$p_{F,t+1}=\frac{p_{F,t}p_{M,t}+\frac{1}{2}\left(1-c\right)\left(1-hs\right)\left(p_{F,t}q_{M,t}+q_{F,t}p_{M,t}\right)}{w_{t}},$$

and

(7) $$q_{F,t+1}=\frac{\left(1-s\right)q_{F,t}q_{M,t}+\frac{1}{2}\left(1+c\right)\left(1-hs\right)\left(p_{F,t}q_{M,t}+q_{F,t}p_{M,t}\right)}{w_{t}},$$

where

(8) $$w_{t}=p_{F,t}p_{M,t}+\left(1-hs\right)\left(p_{F,t}q_{M,t}+p_{M,t}q_{F,t}\right)+\left(1-s\right)q_{F,t}q_{M,t}.$$

Based on the equations above, we evaluated the independent and interactive effects on driver allele frequency (*q* = mean of *q*_M_ and *q*_F_ for haplodiploids) of zygote versus germline conversion, *c*, *s, h,* and *q*_0_, the initial frequency of the driver allele (i.e., at generation 0) in both sexes. Unless noted otherwise, we set *q*_0_ = 0.001. In 10 previous papers with evolutionary models of CRISPR gene drive in diploids, *q*_0_ was 0.00001 (Unckless et al., 2017), 0.001 (Godfray et al., 2017; Unckless et al., 2015), 0.01 (Champer et al., 2018; Deredec et al., 2011; Noble et al., 2017), 0.05 (Deredec et al., 2008; Hammond et al., 2016), 0.1 (Rode et al., 2019), 0.6 (Rode et al., 2019), and 0.7 (Drury et al., 2017). In one previous analysis, *q*_0_ varied from ca. 0.002 to 0.1 (Figure S4 of Unckless et al., 2017).

To map the outcomes for the driver allele (fixed, lost, fixed or lost depending on the initial driver allele frequency, or stable polymorphism) across a range of parameter values, we solved the equations above for equilibria as described in Supporting Information S1. We also used sensitivity analyses by varying parameters systematically in computer simulations, as described below.

### 2.3 Models with resistance to drive

We modeled the evolutionary dynamics of the CRISPR gene drive with the potential for evolution of resistance to drive under two scenarios. In the first scenario, we examined evolution of resistance to drive using Equations (1), (2), (3) of Unckless et al. (2017) for diploids with zygote conversion and modifying these equations to represent haplodiploids as described below. We set the initial frequency of the resistance and driver alleles at 0.001, with *h* = 0.5, *s = *0.2, no fitness cost for the resistance allele, and *c = *0.9 or 0.5. With this low fitness cost of the driver allele (*s = *0.2), the results for zygote and germline conversion are similar. In the second scenario, we focused exclusively on the rate of decrease in the driver allele frequency caused by the resistance to drive. We simulated initial frequencies of 0.999 for the driver allele, 0.001 for the resistance allele, and 0 for the wild‐type allele. In this scenario, no conversion from wild‐type allele to driver allele occurs, which means the results are independent of the timing of conversion.

To modify the diploid equations of Unckless et al. (2017) to represent haplodiploids, we let *s_r_* be the fitness cost of the resistance allele relative to the wild‐type allele with 0 ≤ *s_r_* <s, and *h_ro_* the dominance of this fitness cost in resistance/wild‐type allele heterozygotes. Thus, the fitness cost in resistance/wild‐type heterozygotes is *h_ro_s_r_*. We let *h_rd_* be the dominance of the fitness advantage of the resistance allele relative to the driver allele in resistance/driver heterozygotes, which makes this fitness advantage *h_rd_s_r_* + (1 − *h_rd_*)*s*. For haplodiploids in generation *t*, we denote the frequencies of the wild‐type, driver, and resistance alleles in males by *p_M,t_*, *q_M,t_* and *r_M,t_*, and their counterparts in females by *p_F,t_*, *q_F,t_*, and *r_F,t_*. In generation *t + 1*, these frequencies are as follows:

(9) $$p_{M,t+1}=\frac{p_{F,t}}{p_{F,t}+q_{F,t}\left(1-s\right)+r_{F,t}\left(1-s_{r}\right)},$$

(10) $$q_{M,t+1}=\frac{q_{F,t}\left(1-s\right)}{p_{F,t}+q_{F,t}\left(1-s\right)+r_{F,t}\left(1-s_{r}\right)},$$

(11) $$r_{M,t+1}=\frac{r_{F,t}\left(1-s_{r}\right)}{p_{F,t}+q_{F,t}\left(1-s\right)+r_{F,t}\left(1-s_{r}\right)},$$

(12) $$p_{F,t+1}=\frac{1}{w_{t}}\times \left\{\begin{array}{c} \begin{array}{cc} & p_{F,t}p_{M,t}+\frac{1}{2}\left(1-c\right)\left(p_{F,t}q_{M,t}+p_{M,t}q_{F,t}\right)\left(1-hs\right)\\ & \;+\frac{1}{2}\left(p_{M,t}r_{F,t}+p_{F,t}r_{M,t}\right) \end{array}\\ \left(1-h_{\mathrm{ro}}s_{r}\right) \end{array}\right\},$$

(13) $$\begin{array}{cc} q_{F,t+1} & =\frac{1}{w_{t}}\times \left[\left\{q_{F,t}q_{M,t}+c\left(p_{F,t}q_{M,t}+p_{M,t}q_{F,t}\right)\right\}\left(1-s\right)\right.\\ & \;\;\;+\frac{1}{2}\left(1-c\right)\left(p_{F,t}q_{M,t}+p_{M,t}q_{F,t}\right)\left(1-hs\right)\\ & \;\;\;\left.+\frac{1}{2}\left(q_{M,t}r_{F,t}+q_{F,t}r_{M,t}\right)\left\{1-h_{\mathrm{rd}}s_{r}-\left(1-h_{\mathrm{rd}}\right)s\right\}\right] \end{array}$$

(14) $$\begin{array}{cc} r_{F,t+1} & =\frac{1}{w_{t}}\left[r_{F,t}r_{M,t}\left(1-s_{r}\right)+\frac{1}{2}\left(p_{M,t}r_{F,t}+p_{F,t}r_{M,t}\right)\left(1-h_{\mathrm{ro}}s_{r}\right)\right.\\ & \;\;\left.+\frac{1}{2}\left(q_{M,t}r_{F,t}+q_{F,t}r_{M,t}\right)\left\{1-h_{\mathrm{rd}}s_{r}-\left(1-h_{\mathrm{rd}}\right)s\right\}\right] \end{array}$$

where

(15) $$\begin{array}{cc} w_{t} & =p_{F,t}p_{M,t}+\left(1-c\right)\left(p_{F,t}q_{M,t}+p_{M,t}q_{F,t}\right)\left(1-hs\right)\\ & \;+\left\{q_{F,t}q_{M,t}+c\left(p_{F,t}q_{M,t}+p_{M,t}q_{F,t}\right)\right\}\left(1-s\right)\\ & \;+\left(p_{M,t}r_{F,t}+p_{F,t}r_{M,t}\right)\left(1-h_{\mathrm{ro}}s_{r}\right)+\left(q_{M,t}r_{F,t}+q_{F,t}r_{M,t}\right)\\ & \;\times \left\{1-h_{\mathrm{rd}}s_{r}-\left(1-h_{\mathrm{rd}}\right)s\right\}+r_{F,t}r_{M,t}\left(1-s_{r}\right). \end{array}$$

### 2.4 Computer simulations

We conducted computer simulations in R by iterating the relevant equations above for haplodiploids and diploids. In simulations without the potential for resistance to the drive, we simulated enough generations with each set of parameters to classify the outcome for the driver allele as fixed, lost, or stable polymorphism (0 < *q* < 1). In nearly all cases, this was < 100 generations. For simulations where the driver allele increased toward fixation, we recorded the number of generations for *q* to exceed 0.50 (*g*_50_) to compare the rate of increase between haplodiploids and diploids and to systematically assess the effects of *c*, *s, h, and q*_0_, and zygote versus germline conversion, and their interactions within each genetic system.

In simulations with the potential for resistance to the drive, we evaluated two sets of initial conditions: 1) *q*_0_ = *r*_0_ = 0.001 (where *r*_0_ is the initial frequency of the allele conferring resistance to drive) and 2) *q*_0_ = 0.999 and *r*_0_ = 0.001. We simulated enough generations to determine when frequency of the resistance allele exceeded 0.50 (which also indicated *q* was less than 0.50). This was < 200 generations in nearly all cases.

### 2.5 Comparisons with previously published modeling results

For diploid modeling of gene drive, our results from simulations and analyses of equilibria corresponded precisely with results based on the same conditions reported by Deredec et al. (2008), Unckless et al. (2015), Unckless et al. (2017), and Rode et al. (2019). For haplodiploid modeling without gene drive, our results from simulations matched those of Crowder and Carrière (2009).

## 3 RESULTS

### 3.1 Evolutionary trajectories without resistance to drive

**FIGURE 2:**
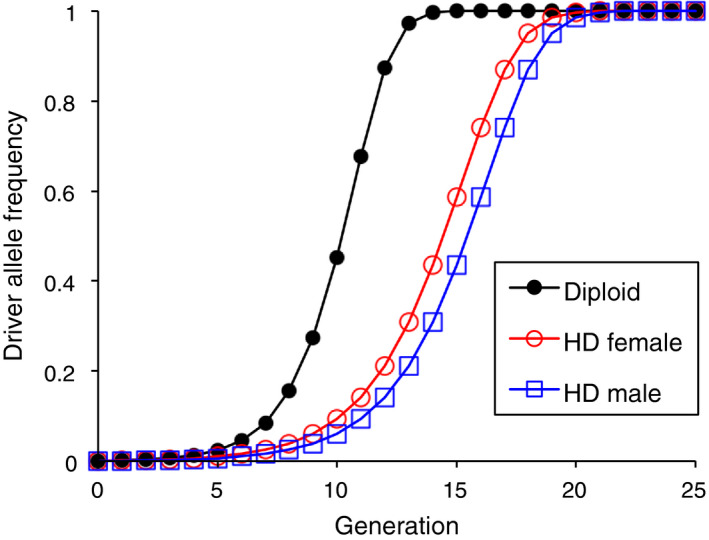
Driver allele frequency increases slower in haplodiploid (HD) than diploid species. No fitness cost (*s* = 0), conversion rate 90% (*c* = 0.9), and initial driver allele frequency (*q*_0_) = 0.001

We first examined the evolutionary trajectories of the CRISPR gene drive with the initial driver allele frequency (*q_0_*) of 0.001, conversion of 90% of heterozygotes to driver allele homozygotes (*c* = 0.9), no effect of the driver allele on fitness (*s* = 0), and without the potential for evolution of resistance to the drive. Under these conditions, the trajectories leading to fixation of the driver allele were qualitatively similar for the haplodiploid and diploid species, but the driver allele spread slower for the haplodiploids (Figure 2). The number of generations for the driver allele frequency to exceed 0.50 (*g*_50_) was 15 for the haplodiploid versus 11 for the diploid species (Figure 2). Because the driver allele had no effect on fitness in this scenario, the 36% greater *g*_50_ for the haplodiploids can be attributed entirely to the lack of conversion in haplodiploid males. In the haplodiploids, the driver allele frequency lagged one generation behind in the haploid males relative to the diploid females (Figure 2). Because the driver allele had no effect on fitness (*s* = 0), the results for this scenario are not affected by the timing of conversion (see below).

**FIGURE 3:**
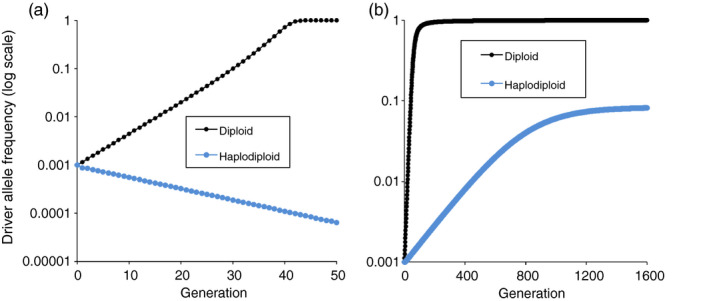
Conditions for increase in driver allele frequency are more restrictive for haplodiploid than diploid species. Initial driver allele frequency *q*_0_ = 0.001 with conversion in zygotes. (a) Fitness cost (*s*) = 0.4, conversion rate (*c*) = 0.9, dominance of fitness cost (*h*) = 0.5. (b) *s = c* = 0.2, *h* = 0

Next, we evaluated the same conditions as above, but with zygote conversion and a substantial fitness cost associated with the driver allele (*s = *0.4) that was codominant (*h* = 0.5). For the diploids, this fitness cost slowed the spread of the driver allele, but the driver allele still increased to fixation (*g*_50_ = 39, Figure 3a). By contrast, in the haplodiploids, the driver allele frequency decreased, ultimately leading to the expected loss of this allele (Figure 3a). With a conversion efficiency of only 20% (*c* = 0.2) and a smaller, recessive fitness cost (*s* = 0.2, *h* = 0), the driver allele frequency increased to fixation in the diploid (*g*_50_ = 68), whereas it reached a stable equilibrium of 0.082 in the haplodiploid (Figure 3b).

**FIGURE 4:**
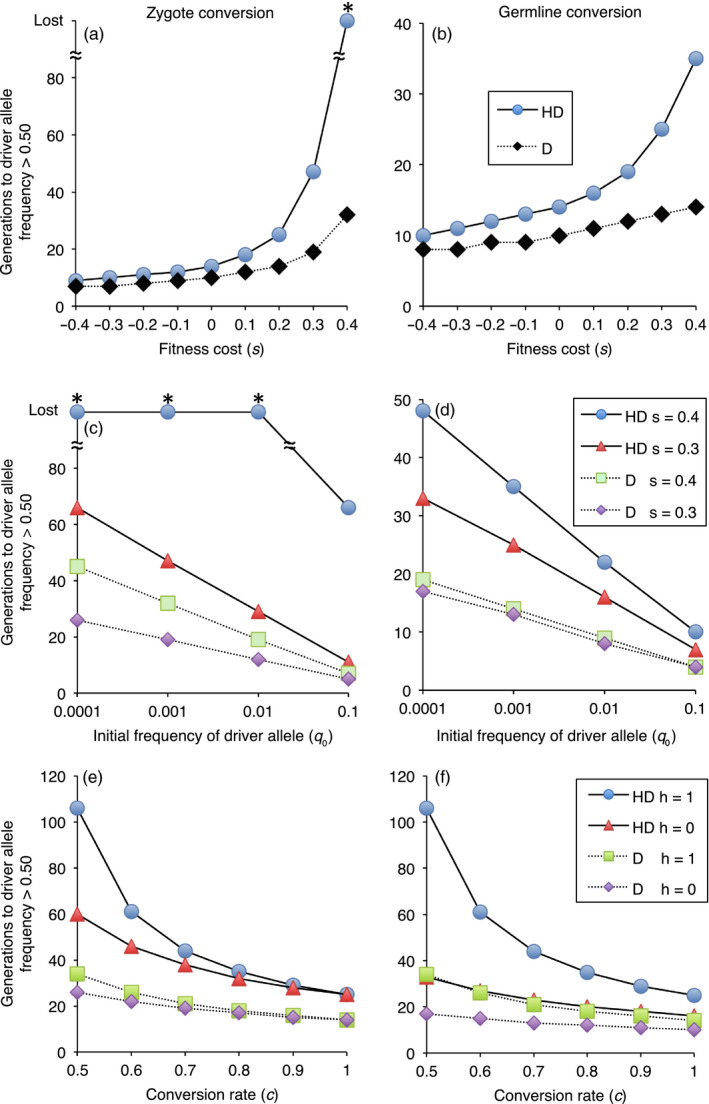
Effects of fitness cost (*s*), initial frequency of the driver allele (*q_0_*), conversion rate (*c*), and dominance of the fitness cost (*h*) on the number of generations for the driver allele frequency to exceed 0.50 in haplodiploid (HD) and diploid (D) species. Panels (a) and (b): *c* = 1, *h = *0.5, *q_0_* = 0.001. Panels (c) and (d): *c* = 1, *h = *0.5. Panels (e) and (f): *s* = 0.2, *q_0_* = 0.001. With *c = *1 and zygote conversion, as in (a) and (c), all heterozygotes are converted to driver allele homozygotes, so the dominance of the fitness cost does not affect the outcome. The boxed keys for germline conversion (b, d, and f) also apply for zygote conversion (a, c, and e, respectively). Asterisks indicate loss of the driver allele, which occurs with *s* > 0.382 in (a) and *q_0_* ≤ 0.0857 in (c)

We conducted a sensitivity analysis with 100% conversion (*c = *1) at either the zygote or germline stage and a fitness cost (*s*) that varied from −0.4 to 0.4, where negative values indicate a fitness benefit of the driver allele (e.g., for a rescue drive in a valued species). With *c = *1 and zygote conversion (Figure 4a,c), all heterozygotes are converted to driver allele homozygotes, so the dominance of the fitness cost (*h*) does not affect the outcome. With germline conversion, the outcome depends on *h* and we evaluated *h = *0.5 in this analysis. For any given fitness cost, the driver allele spread slower for the haplodiploid than the diploid species, and this difference increased as the fitness cost increased (Figure 4a,b). With *s* = −0.4, the *g*_50_ was only two generations more for haplodiploids than diploids with either zygote conversion (9 versus 7) or germline conversion (10 versus 8, Figure 4a,b). With *s = *0.4 and zygote conversion, the driver allele was lost for the haplodiploids and *g*_50_ was 32 for the diploids (Figure 4a). Because the conversion rate was 100% (the maximum), the results indicate that under the conditions evaluated, the fitness cost must be less than 0.4 for the driver allele to increase in haplodiploids. With *s = *0.4 and germline conversion, *g*_50_ was 2.5‐fold greater for haplodiploids (35) than diploids (14) (Figure 4b).

As we increased the initial frequency of the driver allele (*q_0_*) from 0.0001 to 0.1, the driver allele spread more readily (Figure 4c,d). Furthermore, for each combination of *q_0_* and fitness cost examined (*s = *0.3 or 0.4), the driver allele spread more readily in the diploid than the haplodiploid species. With *s = *0.3, the driver allele frequency increased slower in haplodiploids than diploids (Figure 4c,d). With *s = *0.4 and zygote conversion, the driver allele frequency increased in all cases for diploids, but for haplodiploids it increased only for *q_0_* = 0.1 and decreased for *q_0_* = 0.01 or less (Figure 4c). This frequency dependence is important, because in most cases, the driver allele is expected to be rare initially.

With *q_0_* = 0.001, *s* = 0.2, and *h* = 0 or 1, increasing *c* from 0.5 to 1 made the driver allele spread faster (Figure 4e,f). For each combination of parameter values tested, the driver allele spread slower in the haplodiploid than the diploid species (Figure 4e,f). For each combination of *c* and *s*, the driver allele spread faster when the fitness cost was recessive (*h* = 0) than dominant (*h* = 1), with the exception that results were independent of *h* for *c* = 1 with zygote conversion, as noted above. The effect of dominance of the fitness cost was strongest at the lowest *c* and stronger for haplodiploids than diploids (Figure 4e,f).

The effects of timing of conversion (zygote versus germline) depended on the fitness cost. With a fitness cost that is not completely dominant (*s* > 0 and *h* < 1), the driver allele spread slower with zygote conversion (Figure 4a,c,e) than germline conversion (Figure 4b,d,f). This difference arises because the fitness cost occurs throughout the lifetime of the heterozygotes in which conversion to driver allele homozygotes happens at the zygote stage, but not the germline stage. With a dominant fitness cost (*s* > 0 and *h* = 1), the results are identical for conversion at the zygote and germline stages, because the full fitness cost of the driver allele occurs throughout the lifetime of heterozygotes, regardless of the timing of conversion (Figure 4e,f). With no fitness cost (*s* = 0), results are also identical for zygote and germline conversion (Figure 4a,b). With a fitness benefit (*s* < 0), the driver allele spread faster with zygote conversion than germline conversion, because the fitness benefit begins sooner with zygote conversion (Figure 4a,b). However, under the conditions we tested, this difference was small, even when the fitness benefit was relatively large. For example, with *s* = −0.4 and *h* = 0.5, *g*_50_ for haplodiploids was 9 with zygote conversion versus 10 with germline conversion (Figure 4a,b).

**FIGURE 5:**
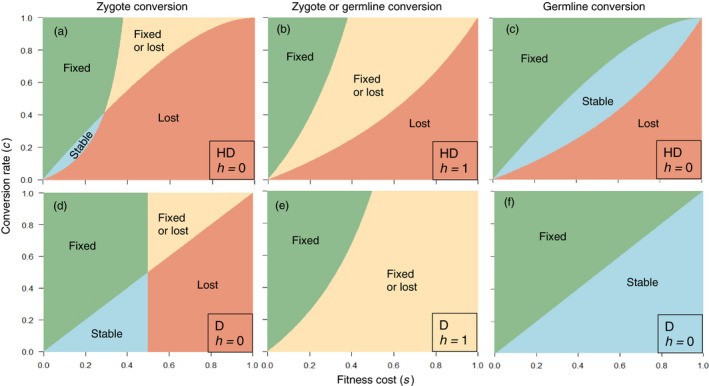
Effects of fitness cost (*s*) and conversion rate (*c*) on driver allele outcomes in haplodiploid and diploid species. The four outcomes for the driver allele are fixed (*q* = 1), lost (*q* = 0), fixed or lost depending on initial driver allele frequency, and stable polymorphism where both the driver and wild‐type alleles persist. Compare each top panel for haplodiploid (HD: panels a to c) with the panel immediately below for diploid (D: panels d to f, adapted from Deredec et al., 2008), with all other conditions identical in each pair. a and d: dominance of the fitness cost (*h*) = 0, conversion in zygote. b and e: *h* = 1, conversion in zygote or germline. c and f: *h* = 0, conversion in germline

Overall, for both haplodiploids and diploids, we can classify driver allele outcomes into four categories: fixed (*q = *1), lost (*q = *0), fixed or lost depending on the initial driver allele frequency (*q_0_*), and stable equilibrium (0 < *q* < 1). By analyzing the equations underlying the evolutionary dynamics (Supporting Information S1), we determined which outcomes occur as a function of all possible values of conversion rate (*c* = 0 to 1) with the fitness cost (*s*) ranging from 0 to 1 and being either recessive (*h* = 0) or dominant (*h* = 1), and with either zygote or germline conversion (Figure 5). As expected for both haplodiploids and diploids, fixation of the driver allele is more likely as *c* increases and *s* decreases (Figure 5). Consistent with the simulation results described above, this analysis demonstrates that the conditions causing fixation of the driver allele are narrower for haplodiploids than diploids, whereas a wider range of conditions yield loss of the driver allele for haplodiploids than diploids (Figure 5). For example with *c* = 1 and conversion at the zygote stage, the driver allele increases to fixation for *q_0_* ≥ 0 with *s* < 0.382 for haplodiploids and *s* < 0.5 for diploids (Supporting Information S1). For both haplodiploids and diploids, stable equilibria occurred with a recessive fitness cost (*h* = 0), but not with a dominant fitness cost (*h* = 1, Figure 5). Fixation of the driver allele is expected when some conversion occurs (*c* > 0) and a fitness benefit is associated with the driver allele (*s* < 0), because both conversion and selection favor the driver allele (Supporting Information S1).

Consistent with the simulation results described above, with a dominant fitness cost (*h* = 1), the results are identical for zygote and germline conversion (Figure 5b,e). However, with a recessive fitness cost (*h* = 0), conditions favoring fixation or stability of the driver allele are narrower with zygote conversion (Figure 5a,d) than germline conversion (Figure 5c,f). For example, with *c* = 1 and *h* = 0 for haplodiploids, fixation of the driver allele occurs with *s < *0.382 (as noted above) for zygote conversion versus *s < *1 for germline conversion (Figure 5a,c and Supporting Information S1).

### 3.2 Evolutionary trajectories with resistance to drive

**FIGURE 6:**
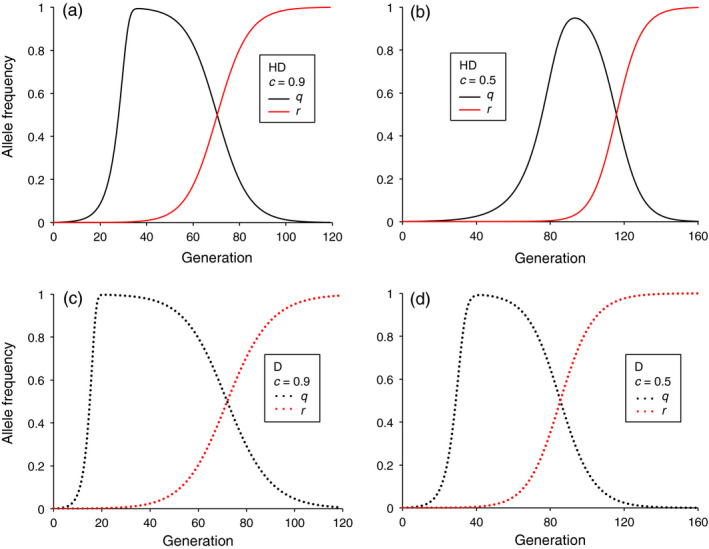
After the driver allele reaches a high frequency, resistance to the drive evolves faster in haplodiploid than diploid species. Frequency of driver allele (*q*, black) and resistance allele (*r*, red). Initial frequency of driver allele (*q*_0_) and resistance allele (*r*_0_) = 0.001, driver allele fitness cost (*s*) = 0.2 and dominance of fitness cost (*h*) = 0.5, no fitness cost for resistance allele, and fitness cost for resistance/driver heterozygotes = 0.1. Compare each top panel for haplodiploid (a and b, solid lines) with the panel immediately below for diploid (c and d, dotted lines), with all other conditions identical in each pair

**FIGURE 7:**
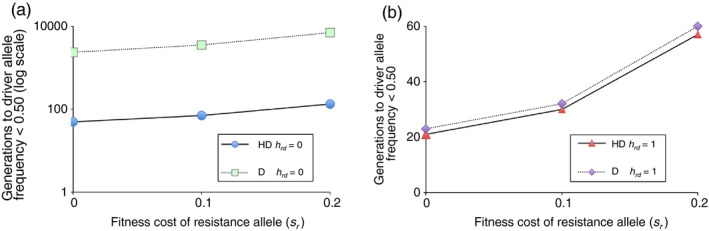
Effects of fitness cost of the resistance allele (*s*_r_) on evolution of resistance to drive in haplodiploid (HD) and diploid (D) species. The y‐axis shows the number of generations for the driver allele frequency (*q*) to decrease below 0.50 as the frequency of the resistance allele (*r*) increases. *q*_0_ = 0.999, *r*_0_ = 0.001. Fitness cost of driver allele (*s*) = 0.3. Fitness advantage of the resistance allele relative to the driver allele in resistance/driver heterozygotes either (a) recessive (*h*_rd_ = 0) or (b) dominant (*h*_rd_ = 1)

In our final set of simulations, we included the potential for evolution of resistance to the drive, mediated by an allele at the target locus that could not be converted to the driver allele. We analyzed situations where resistance alleles occurred initially at a low frequency as part of standing genetic variation and selection favored the resistance allele relative to the driver allele, but not relative to the wild‐type allele. Thus, the fitness cost of the resistance allele (*s_r_*) was less than the fitness cost of the driver allele (0 ≤ *s_r_* < s). Accordingly, selection favoring the resistance allele was weak when the driver allele was rare. After the driver allele reached a high frequency via conversion of the wild‐type allele, selection increased the resistance allele frequency and concomitantly decreased the driver allele frequency (Figure 6). In all cases examined, resistance to the drive evolved faster in haplodiploids than diploids (Figures 6 and 7).

In the first scenario with resistance to drive, we set the initial frequency of the resistance and driver alleles at 0.001, with a codominant (*h* = 0.5) fitness cost of 0.2 for the driver allele, no fitness cost for the resistance allele, *c = *0.9, and zygote conversion. Consistent with the results without resistance summarized above, the driver allele frequency increased slower in the haplodiploids (*g*_50_ = 28) than the diploids (*g*_50_ = 15); it peaked at 0.993 in generation 37 for the haplodiploids versus 0.997 in generation 21 in the diploids (Figure 6a,c). The number of generations for the driver allele frequency to decline from its peak to less than 0.50 was 34 for the haplodiploids versus 52 for the diploids (Figure 6a,c). Under the conditions tested initially, the addition of the resistance allele had little effect on the upward trajectory of the driver allele. In simulations with and without the resistance allele, the *g*_50_ was the same. The driver allele frequency was slightly higher without resistance than with resistance in the generation when the threshold of 0.50 was exceeded (generation 28: 0.509 without resistance versus 0.505 with resistance in the haplodiploids and generation 15: 0.503 without resistance versus 0.5004 with resistance in the diploids).

With *c* = 0.5 and all other conditions as stated immediately above, the driver allele frequency increased slower in the haplodiploid (*g*_50_ = 76) than the diploid (*g*_50_ = 29) and reached a lower peak in the haplodiploids (0.949 at generation 93) than the diploids (0.991 at generation 44, Figure 6b,d). Under this scenario, the number of generations for the driver allele frequency to decline from its peak to less than 0.50 was 23 for the haplodiploids and 42 for the diploids (Figure 6b,d). Reflecting the rate of the increase in driver allele frequency and its subsequent decrease, the number of generations when the driver allele frequency was above 0.50 was 35 to 42% greater for diploids than haplodiploids (Figure 6, *c* = 0.9, haplodiploid = 43 and diploid = 58; *c* = 0.5, haplodiploid = 40 and diploid = 57).

To simplify the dynamics and focus exclusively on the rate of decrease in the driver allele frequency caused by the resistance to drive, we simulated initial frequencies of 0.999 for the driver allele, 0.001 for the resistance allele, and 0 for the wild‐type allele. In this scenario, no CRISPR‐mediated conversion occurs, and the trajectory is determined entirely by the fitness of the three genotypes (driver allele homozygote, resistance allele homozygote, and resistance/driver heterozygote). With a fitness cost (*s*) of 0.3 for the driver allele and the fitness cost of the resistance allele (*s_r_*) varied from 0 to 0.2, the driver allele decreased faster for the haplodiploid than the diploid species (Figure 7).

For both haplodiploids and diploids, the driver allele frequency decreased slower when the fitness advantage of the resistance allele relative to the driver allele was recessive (*h_rd_* = 0, Figure 7a) versus dominant (*h_rd_* = 1, Figure 7b). Moreover, the difference between haplodiploids and diploids was much larger with recessive versus dominant resistance (Figure 7). This occurred because selection favored the resistance allele in haploid males regardless of its dominance, but not in heterozygous diploids when this allele was recessive. Specifically, with *h_rd_* = 0 and *s_r_* ranging from 0 to 0.2, the number of generations for the driver allele frequency to drop below 0.50 was 2,352 to 7,041 generations for the diploids versus 49 to 133 for the haplodiploids (Figure 7a). This is 48‐ to 53‐fold more generations for the diploids than the haplodiploids. Conversely, with the fitness advantage of the resistance allele relative to the driver allele dominant (*h_rd_* = 1) and *s_r_* varied from 0 to 0.2, the generations for the driver allele frequency to decrease below 0.50 was 23 to 60 for the diploids versus 21 to 57 for the haplodiploids, less than a 1.1‐fold increase for the diploids relative to haplodiploids (Figure 7b).

## 4 DISCUSSION

In contrast with the extensive empirical and theoretical studies of CRISPR gene drive in diploid organisms, relatively little is known about this issue in haplodiploids despite their immense global impacts. Collectively, the myriad species of haplodiploids have devastating negative effects as pests in agriculture and invasive species in native habitats, as well as vital positive contributions as pollinators and natural enemies of pests (Biedermann et al., 2019; Crowder & Carrière, 2009; De Barro et al., 2011; He et al., 2020; Macfadyen et al., 2018; McLaughlin & Dearden, 2019; Normark, 2003; Van Leeuwen et al., 2015). The results reported here show that in principle, CRISPR driver alleles can spread in wild populations of haplodiploids across a wide range of conditions. Indeed, qualitative patterns were generally similar between haplodiploids and diploids for the spread of alleles via gene drive and the evolution of resistance to gene drive. However, the conditions favoring spread of driver alleles were narrower in haplodiploids than diploids. Also, for each set of parameter values tested, the driver allele frequency increased slower and resistance to the drive evolved faster in haplodiploids than diploids.

The extent of the difference in evolutionary dynamics between haplodiploids and diploids was affected by nonlinear interactions among the factors examined, including the magnitude (*s*) and dominance (*h*) of the fitness cost or benefit associated with the driver allele, conversion efficiency (*c*), the initial frequency of the driver allele (*q*_0_), and the timing of conversion. For example, with *s = *0.4, *h* = 0.5, *c = *0.9, and *q*_0_ = 0.001, and zygote conversion, outcomes were diametrically opposite: loss of the driver allele in the haplodiploids and fixation in the diploids. However, with a sufficiently lower *s* or higher *q*_0_, the outcomes were similar: the driver allele increased to fixation in both the haplodiploid and diploid species, only slower in the haplodiploids.

Here, we focused primarily on a low initial frequency of the driver allele (e.g., *q*_0_ = 0.001) because we expect that in most applications of gene drive, a relatively small number of laboratory‐reared organisms with the driver allele will be released into large field populations. When the driver allele is rare initially, its spread can be prevented by fitness costs. As *q*_0_ approaches zero and the fitness cost is dominant (*h* = 1), even with 100% conversion efficiency, the driver allele can spread only if the fitness cost is less than 0.5 for diploids or less than 0.382 for haplodiploids, with either zygote or germline conversion.

The timing of conversion did not affect results with *s* = 0 or *h = *1, but with *s > *0 and *h < *1, the spread of the driver allele was slower and occurred under a narrower range of conditions with zygote conversion than germline conversion. In the few species of diploids rigorously examined so far, germline conversion is more important than zygote conversion (Champer et al., 2017; Grunwald et al., 2019; Hammond et al., 2017). Thus, with *s > *0 and *h < *1, results with germline conversion are probably more realistic, whereas results with zygote conversion provide a conservative assessment of the potential spread of driver alleles.

In haplodiploids as well as in diploids, the potential for evolution of resistance to gene drives represents a major challenge, with some promising countermeasures identified in theoretical and empirical work with diploids (Champer et al., 2018 and 2020; Drury et al., 2017; Godfray et al., 2017; Noble et al., 2017; Unckless et al., 2017). Relative to the results here with alleles conferring resistance to drive considered only from standing genetic variation (initial frequency = 0.001), the addition of resistance alleles arising from nonhomologous end‐joining during CRISPR‐mediated cleavage and other mechanisms could substantially accelerate evolution of resistance to drive under some conditions (Unckless et al., 2017). Moreover, we found that resistance to drive evolved faster in haplodiploids than diploids, with a much larger difference between the two genetic systems when the fitness advantage of the resistance allele relative to the driver allele was recessive. As shown before for diploids (Drury et al., 2017; Godfray et al., 2017) and seen here for haplodiploids, the magnitude and dominance of the fitness advantage of alleles conferring resistance to drive relative to driver alleles determines if and how quickly resistance alleles spread. Thus, driver alleles with minimal fitness cost can help to deter evolution of resistance to drive.

In light of the results summarized above, it may be most productive to aim for population replacement for haplodiploid pests rather than suppression or eradication. This approach entails introducing traits that have little or no fitness cost, but reduce the harm caused by target populations. For pests such as whiteflies and thrips that cause extensive damage to crops by transmitting plant viruses, this idea might be implemented with alleles that reduce such transmission without decreasing the insects' fitness. For example, introducing an allele that interferes with *B. tabaci* transmission of begomoviruses, which damage many important crops (Mansoor, Briddon, Zafar, & Stanley, 2003), might boost this pest's fitness by reducing the negative effects of the viruses on the vector that occur in some cases (Costa, Brown, & Byrne, 1991; Liu, Zhao, Jiang, Zhou, & Liu, 2009; Rubinstein & Czosnek, 1997). This tactic is analogous to reducing the transmission by mosquitoes of pathogens that cause malaria and other human diseases without necessarily reducing the mosquitoes' fitness.

Another potential application that could succeed in haplodiploids as well as in diploids is increasing the frequency of alleles conferring pest susceptibility to conventional insecticides or insecticidal transgenic crops (Itokawa, Komagata, Kasai, Ogawa, & Tomita, 2016; Jin et al., 2018). In both haplodiploids and diploids, efforts to increase the frequency of susceptible alleles via gene drive would be aided by the fitness cost typically associated with insecticide resistance alleles (Crowder & Carrière, 2009; Gassmann, Carrière, & Tabashnik , 2009; Kliot & Ghanim, 2012). Moreover, the use of CRISPR‐mediated gene drive to spread traits that increase the fitness of valued species, such as providing resistance to parasitic mites or pesticides in bees (Belsky & Joshi, 2019; Hu, Zhang, Liao, & Zeng, 2019), should be boosted by the fitness benefits of the driver alleles. Our results imply the spread of fitness‐enhancing alleles would not be greatly hindered in haplodiploids relative to diploids. Because such alleles typically carry fitness costs in the absence of the selective agents such as mites or insecticides, selection would only favor such alleles when populations suffer the detrimental effects of these selective agents. However, CRISPR‐mediated spread of such alleles could effectively immunize beneficial populations and thereby minimize the negative effects of such selection.

The ethical concerns about gene drive in diploids (Brossard, Belluck, Gould, & Wirz, 2019; de Graeff, Jongsma, Johnston, Hartley, & Bredenoord, 2019; James, Marshall, Christophides, Okumu, & Nolan, 2020; Kohl, Brossard, Scheufele, & Xenos, 2019; Scott et al., 2018; Webber, Raghu, & Edwards, 2015) also apply to haplodiploids, perhaps tempered somewhat for haplodiploids by the expected greater difficulty in spreading driver alleles, increased likelihood of evolution of resistance to the drive, and reduced chances of population control or eradication. Although the exploration of gene drive in haplodiploids is in its infancy, we hope the theoretical framework provided here will spur progress, including additional modeling as well as empirical advances. The results here also provide insights into potential differences in the evolutionary trajectories of gene drives targeting sex‐linked versus autosomal loci in diploids.

## CONFLICT OF INTEREST

P.W.A. and L.L.W. have a U.S. Provisional Patent Application No. 62/734,953: Method for Genetic Manipulation of Sap‐Feeding Insects. The other authors have no conflicts of interest.

## AUTHOR CONTRIBUTIONS

BET, JL, and SM conceived the study. JL created the simulation models and analyzed the equations underlying the evolutionary dynamics. JL and BET ran simulations and analyzed the results. BET and JL wrote the manuscript with contributions from all authors.

## Supporting information

Supplementary Material

## Data Availability

The simulation models used in this study are available through Dryad https://doi.org/10.5061/dryad.mw6m905tk.
