# Supplementary material for: Can CRISPR gene drive work in pest and beneficial haplodiploid species?
Source: Evol Appl. 2020 Jun 19;13(9):2392–403. doi: 10.1111/eva.13032 (PMC7513724; doi:10.1111/eva.13032)
Supplement: Supplementary file 1 — Supplementary Material [file EVA-13-2392-s001.pdf]

Supporting Information

Can CRISPR gene drive work in pest and beneficial haplodiploid species?

Jun Li,[1] Ofer Aidlin Harari,[2] Anna-Louise Doss,[3] Linda L. Walling,[4]

Peter W. Atkinson,[3] Shai Morin,[2] Bruce E. Tabashnik[5]*

[1] Department of Statistics, University of California, Riverside, CA 92521, USA.

[2] Department of Entomology, Hebrew University of Jerusalem, Rehovot 7610001, Israel.

[3] Department of Entomology, University of California, Riverside, CA 92521, USA.

[4] Department of Botany and Plant Sciences, University of California, Riverside, CA 92521, USA

[5] Department of Entomology, University of Arizona, Tucson, AZ 85721, USA

*Corresponding author. Email: brucet@cals.arizona.edu (B.E.T).

Solving equations for equilibria to map outcomes of gene drive

To map the outcomes for the driver allele (fixed, lost, fixed or lost depending on the initial driver allele frequency, or stable polymorphism) across a range of parameter values in the absence of evolution of resistance to drive, we solved the relevant equations for equilibria as described below. Based on their equation 8, Deredec, Burt & Godfray (2008) developed the following equilibrium conditions for the evolutionary dynamics for diploids with conversion occurring in the zygotes. The equilibrium frequency for the driver allele, q^*, is

$$q^* = 0 \ if \ s > \frac{c}{1-h+ch} \text{ and } s > \frac{c}{2c+h-ch};$$

$$q^* = 1 \ if \ s < \frac{c}{1-h+ch} \text{ and } s < \frac{c}{2c+h-ch}.$$

When these inequalities do not hold, there is an interior equilibrium

$$q^* = q^{interior} = \frac{c(1-2s) - hs(1-c)}{s(1-2c-2h(1-c))}.$$

The above interior equilibrium is stable if

$$\frac{c}{1-h+ch} < s < \frac{c}{2c+h-ch},$$

and unstable if

$$\frac{c}{1-h+ch} > s > \frac{c}{2c+h-ch}.$$

Based on the above equilibrium conditions, for the driver allele to reach the fixation, i.e., $q^* = 1$, no matter what the initial q_0 is, we need c, h and s to satisfy the following inequalities

$$s < \frac{c}{1-h+ch} \text{ and } s < \frac{c}{2c+h-ch}.$$

When c =1, the above inequalities reduce to $s < 0.5$.

Similarly, we derived the equilibrium conditions for the evolutionary dynamics of the CRISPR gene drive for haplodiploids as follows. Since $p_{M,t} + q_{M,t} = 1$ and $p_{F,t} + q_{F,t} = 1$ in any generation t, our equations (1)-(4) can be represented by the following two-dimensional discrete dynamical system based on $(q_{M,t}, q_{F,t})$:

$$q_{M,t+1} = \frac{(1-s)q_{F,t}}{(1-q_{F,t})+(1-s)q_{F,t}},$$

$$q_{F,t+1} = \frac{(1-s)q_{F,t}q_{M,t} + \left\{c(1-s) + \frac{1}{2}(1-c)(1-hs)\right\}\{q_{F,t}(1-q_{M,t}) + (1-q_{F,t})q_{M,t}\}}{w_t},$$

where $w_t = (1-q_{F,t})(1-q_{M,t}) + (1-s)q_{F,t}q_{M,t} + \{c(1-s) + (1-c)(1-hs)\}\{q_{F,t}(1-q_{M,t}) + (1-q_{F,t})q_{M,t}\}$.

Define

$$f_1(q_{M,t}, q_{F,t}) = \frac{(1-s)q_{F,t}}{(1-q_{F,t})+(1-s)q_{F,t}},$$

$$f_2(q_{M,t}, q_{F,t}) = \frac{(1-s)q_{F,t}q_{M,t} + \left\{c(1-s) + \frac{1}{2}(1-c)(1-hs)\right\}\{q_{F,t}(1-q_{M,t}) + (1-q_{F,t})q_{M,t}\}}{w_t}.$$

Then the equilibrium values of (q_M^*, q_F^*) are the solutions to the following equations:

$$\begin{cases} q_M = f_1(q_M, q_F) \\ q_F = f_2(q_M, q_F) \end{cases}$$

Solving the above equations yields three solutions:

(a) $q_M^* = q_F^* = 0$;

(b) $q_M^* = q_F^* = 1$;

(c) $q_F^* = \frac{(1+5c+2h-2ch)s-(2c+h-ch)s^2-2c}{2(2h-2hc+2c-1)s+2(1-c)(1-h)s^2}$ and $q_M^* = \frac{q_F^*(1-s)}{(1-q_F^*)+q_F^*(1-s)}$.

According to the results from two-dimensional discrete dynamical systems, the equilibrium (q_M^*, q_F^*) is stable if all the eigenvalues of the Jacobian matrix $\begin{bmatrix} \frac{\partial f_1}{\partial q_M} & \frac{\partial f_1}{\partial q_F} \\ \frac{\partial f_2}{\partial q_M} & \frac{\partial f_2}{\partial q_F} \end{bmatrix}$ at (q_M^*, q_F^*) lie within the unit circle on the complex plane. Besides this eigenvalue requirement, for (q_M^*, q_F^*) in (c) to be a reasonable equilibrium for our dynamical system, they also need to satisfy the following conditions:

$$0 < q_F^* = \frac{(1+5c+2h-2ch)s-(2c+h-ch)s^2-2c}{2(2h-2hc+2c-1)s+2(1-c)(1-h)s^2} < 1 \text{ and } 0 < q_M^* = \frac{q_F^*(1-s)}{(1-q_F^*)+q_F^*(1-s)} < 1.$$

Based on all the conditions described above, we obtain the following results for the three equilibria listed in (a)-(c). The equilibrium frequencies for the drive allele in females and males, q_F^* and q_M^*, are

$$q_F^* = q_M^* = 0 \ \ if \begin{cases} (1+5c+2h-2ch)s-(2c+h-ch)s^2 > 2c \\ \text{and} \\ (3+c+2ch-2h)s-(ch-h+2)s^2 > 2c \end{cases};$$

$$q_F^* = q_M^* = 1 \ \ if \begin{cases} (1+5c+2h-2ch)s-(2c+h-ch)s^2 < 2c \\ \text{and} \\ (3+c+2ch-2h)s-(ch-h+2)s^2 < 2c \end{cases}.$$

When these inequalities do not hold, there is an interior equilibrium

$$q_F^* = \frac{(1+5c+2h-2ch)s-(2c+h-ch)s^2-2c}{2(2h-2hc+2c-1)s+2(1-c)(1-h)s^2} \text{ and } q_M^* = \frac{q_F^*(1-s)}{(1-q_F^*)+q_F^*(1-s)}.$$

The above interior equilibrium is stable if

$$(1+5c+2h-2ch)s-(2c+h-ch)s^2 < 2c < (3+c+2ch-2h)s-(ch-h+2)s^2,$$

and unstable if

$$(1+5c+2h-2ch)s-(2c+h-ch)s^2 > 2c > (3+c+2ch-2h)s-(ch-h+2)s^2.$$

For the driver allele to reach fixation, i.e., $q_F^* = q_M^* = 1$ with any value of q_0 is, c, h and s must satisfy the following inequalities

$$\begin{cases} (1+5c+2h-2ch)s-(2c+h-ch)s^2 < 2c \\ \text{and} \\ (3+c+2ch-2h)s-(ch-h+2)s^2 < 2c \end{cases}.$$

When c =1, the above inequalities reduce to

$$s^2-3s+1 > 0 \text{ and } s^2-2s+1 > 0,$$

which is equivalent to

$$s < \frac{3-\sqrt{5}}{2} = 0.382.$$

All of the equations above address conversion that occurs in zygotes. For germline conversion, Deredec *et al.* (2008) analyzed the equilibrium conditions for diploids based on their equation 1, as explained below.

The equilibrium frequency for the driver allele, q^*, is

$$q^* = 0 \ \ if \ s > \frac{c}{1-h+ch} \text{ and } s > \frac{c}{h(1+c)};$$

$$q^* = 1 \ \ if \ s < \frac{c}{1-h+ch} \text{ and } s < \frac{c}{h(1+c)}.$$

When these inequalities do not hold, there is an interior equilibrium

$$q^* = q^{interior} = \frac{c - hs(1+c)}{s(1-2h)}.$$

The above interior equilibrium is stable if

$$\frac{c}{1-h+ch} < s < \frac{c}{h(1+c)},$$

and unstable if

$$\frac{c}{1-h+ch} > s > \frac{c}{h(1+c)}.$$

Based on the above equilibrium conditions, for the driver allele to reach the fixation, i.e., $q^* = 1$, no matter what the initial q_0 is, we need c, h and s to satisfy the following inequalities:

$$s < \frac{c}{1-h+ch} \text{ and } s < \frac{c}{h(1+c)}.$$

When c =1, the above inequalities reduce to

$$\begin{cases} s < \frac{1}{2h} & if\ h > \frac{1}{2} \\ s < 1 & if\ h \le \frac{1}{2} \end{cases}$$

For germline conversion in haplodiploids, our equations (5)-(8) can be represented by the following two-dimensional discrete dynamical system based on $(q_{M,t}, q_{F,t})$:

$$q_{M,t+1} = \frac{(1-s)q_{F,t}}{(1-q_{F,t})+(1-s)q_{F,t}},$$

$$q_{F,t+1} = \frac{(1-s)q_{F,t}q_{M,t} + \frac{1}{2}(1+c)(1-hs)\{q_{F,t}(1-q_{M,t}) + (1-q_{F,t})q_{M,t}\}}{w_t},$$

where $w_t = (1-q_{F,t})(1-q_{M,t}) + (1-hs)\{q_{F,t}(1-q_{M,t}) + (1-q_{F,t})q_{M,t}\} + (1-s)q_{F,t}q_{M,t}$.

We define

$$f_1(q_{M,t}, q_{F,t}) = \frac{(1-s)q_{F,t}}{(1-q_{F,t})+(1-s)q_{F,t}},$$

$$f_2(q_{M,t}, q_{F,t}) = \frac{(1-s)q_{F,t}q_{M,t}+\frac{1}{2}(1+c)(1-hs)\{q_{F,t}(1-q_{M,t})+(1-q_{F,t})q_{M,t}\}}{w_t}.$$

Then the equilibrium values of (q_M^*, q_F^*) are the solutions to the following equations:

$$\begin{cases} q_M = f_1(q_M, q_F) \\ q_F = f_2(q_M, q_F) \end{cases}$$

Solving the above equations yields three solutions:

(a) $q_M^* = q_F^* = 0$;

(b) $q_M^* = q_F^* = 1$;

(c) $q_F^* = \frac{(1+c)(1+2h)s-(1+c)hs^2-2c}{2\{(1-h)s^2-(1-2h)s\}}$ and $q_M^* = \frac{q_F^*(1-s)}{(1-q_F^*)+q_F^*(1-s)}$

Again based on the results from two-dimensional discrete dynamical systems, the equilibrium (q_M^*, q_F^*) is stable if all the eigenvalues of the Jacobian matrix $\begin{bmatrix} \frac{\partial f_1}{\partial q_M} & \frac{\partial f_1}{\partial q_F} \\ \frac{\partial f_2}{\partial q_M} & \frac{\partial f_2}{\partial q_F} \end{bmatrix}$ at (q_M^*, q_F^*) lie within the unit circle on the complex plane. Besides this eigenvalue requirement, for (q_M^*, q_F^*) in (c) to be a reasonable equilibrium for our dynamical system, they also need to satisfy:

$$0 < q_F^* = \frac{(1+c)(1+2h)s-(1+c)hs^2-2c}{2\{(1-h)s^2-(1-2h)s\}} < 1 \text{ and } 0 < q_M^* = \frac{q_F^*(1-s)}{(1-q_F^*)+q_F^*(1-s)} < 1.$$

Based on all the above conditions, we obtain the following results for the three equilibria listed in (a)-(c). The equilibrium frequencies for the drive allele in females and males, q_F^* and q_M^*, are

$$q_F^* = q_M^* = 0 \;\; if \begin{cases} (1+c)(1+2h)s-(1+c)hs^2 > 2c \\ \text{and} \\ (3+c+2ch-2h)s-(ch-h+2)s^2 > 2c \end{cases};$$

$$q_F^* = q_M^* = 1 \;\; if \begin{cases} (1+c)(1+2h)s-(1+c)hs^2 < 2c \\ \text{and} \\ (3+c+2ch-2h)s-(ch-h+2)s^2 < 2c \end{cases}$$

When these inequalities do not hold, there is an interior equilibrium

$$q_F^* = \frac{(1+c)(1+2h)s-(1+c)hs^2-2c}{2\{(1-h)s^2-(1-2h)s\}} \text{ and } q_M^* = \frac{q_F^*(1-s)}{(1-q_F^*)+q_F^*(1-s)}$$

The above interior equilibrium is stable if

$$(1+c)(1+2h)s-(1+c)hs^2<2c<(3+c+2ch-2h)s-(ch-h+2)s^2,$$

and unstable if

$$(1+c)(1+2h)s-(1+c)hs^2>2c>(3+c+2ch-2h)s-(ch-h+2)s^2.$$

For the driver allele to reach fixation, i.e., $q_F^*=q_M^*=1$ with any value of q_0 is, c, h and s must satisfy the following inequalities

$$\begin{cases} (1+c)(1+2h)s-(1+c)hs^2<2c \\ \text{and} \\ (3+c+2ch-2h)s-(ch-h+2)s^2<2c \end{cases}$$

When c =1, the above inequalities reduce to

$$hs^2-(1+2h)s+1>0 \text{ and } s^2-2s+1>0,$$

which is equivalent to

$$\begin{cases} s<\dfrac{1+2h-\sqrt{1+4h^2}}{2h} & if\ h>0 \\ s<1 & if\ h=0 \end{cases}$$
